# Early-Stage Non-Small Cell Lung Cancer: New Challenges with Immune Checkpoint Blockers and Targeted Therapies

**DOI:** 10.3390/cancers16162779

**Published:** 2024-08-06

**Authors:** Pernelle Lavaud, Martina Bortolot, Lodovica Zullo, David O’Reilly, Jarushka Naidoo, Giannis Mountzios, Olaf Mercier, Lizza E. L. Hendriks, Jordi Remon

**Affiliations:** 1 Gustave Roussy, Department of Cancer Medicine, Paris-Saclay University, 114, rue Edouard Vaillant, 94805 Villejuif, Francelodovica.zullo@gustaveroussy.fr (L.Z.); 2 Department of Respiratory Medicine, Maastricht University Medical Centre, GROW School for Oncology and Reproduction, 6229 ER Maastricht, The Netherlands; 3 Department of Medicine (DMED), University of Udine, 33100 Udine, Italy; 4 Medical Oncology, Beaumont Hospital, RCSI University of Health Sciences, D02 YN77 Dublin, Ireland; 5 Fourth Department of Medical Oncology and Clinical Trials Unit, Henry Dunant Hospital Center, 11526 Athens, Greece; 6 Department of Thoracic Surgery, Hôpital Marie Lannelongue, 92350 Le Plessis-Robinson, France; o.mercier@ghpsj.fr

**Keywords:** early-stage NSCLC, alectinib, osimertinib, perioperative, induction, lung cancer

## Abstract

**Simple Summary:**

The current review summarizes the new potential treatment strategies for patients with early-stage non-small cell lung cancer with immunotherapy and targeted therapies and defines the current challenges for making treatment decisions with these approaches in daily practice.

**Abstract:**

The recent advent of tyrosine kinase inhibitors (TKIs) and immune checkpoint blockers (ICBs) in early-stage non-small cell lung cancer (NSCLC) has dramatically modified treatment strategies by improving the prognosis in this setting. Osimertinib and alectinib, both TKIs, have shown significant improvements in outcomes for patients with resected *EGFR*- and *ALK*-positive NSCLC, respectively, changing the standard of care in these subgroups. More recently, the LAURA trial showed the efficacy of osimertinib after chemoradiotherapy in patients with unresectable stage III NSCLC harboring *EGFR* mutations. Numerous trials are still ongoing to investigate neoadjuvant/perioperative TKIs in several oncogene-driven NSCLC. In addition, several ICBs have been tested and approved as adjuvant (atezolizumab and pembrolizumab), neoadjuvant (nivolumab), and perioperative treatments (pembrolizumab) for patients with resectable early-stage NSCLC. Despite these advances, many challenges remain regarding the use of TKIs and ICBs in this setting, including the optimal duration of adjuvant TKI or induction ICB therapy, the role of minimal residual disease to identify patients at high-risk of disease relapse and to guide adjuvant treatment decisions, and the role of adjuvant chemotherapy in resected oncogene-driven NSCLC. Furthermore, potential predictive biomarkers for efficacy are needed to eventually intensify the entire perioperative strategies. This review aims to summarize and discuss the available evidence, the ongoing trials, and the challenges associated with TKI- and ICB-based approaches in early-stage NSCLC.

## 1. Introduction

Among patients diagnosed with non-small cell lung cancer (NSCLC), approximately 50% have localized (stages I and II) or locally advanced (stage III) disease. Surgery remains the cornerstone treatment for patients with stages I and II NSCLC, as well as select cases of stage IIIA NSCLC. Radical surgery with lymph node dissection, aiming for a complete resection, is essential to ensure the best possible prognosis [1,2]. For those who are not eligible for surgery, for example because of compromised pulmonary function, radiotherapy is generally the local treatment of choice.

Historically, adjuvant chemotherapy for fit patients with resected NSCLC with tumor size ≥4 cm or lymph node involvement was recommended because of the data from a pooled analysis from randomized clinical trials (RCTs) [3]. The analysis demonstrated a 5.4% improvement in 5-year overall survival (OS) with adjuvant chemotherapy compared with no adjuvant treatment (HR of death 0.89; 95% CI 0.82–0.96; *p* = 0.005). The effect of chemotherapy was higher in patients with better performance status. However, the benefit varied with stage (test for trend, *p* = 0.04; hazard ratio, HR for stage IA: 1.40; 95% confidence interval (CI): 0.95–2.06; HR for stage IB: 0.93; 95% CI: 0.78–1.10; HR for stage II: 0.83; 95% CI: 0.73–0.95; and HR for stage III: 0.83; 95% CI: 0.72–0.94) [3]. Although neoadjuvant chemotherapy has reported a similar magnitude of disease-free survival (DFS) and OS benefit compared with adjuvant chemotherapy [4] and better treatment compliance (97% with neoadjuvant or induction CT vs. 66% with adjuvant approach) [5], physicians have preferred adjuvant treatment for several years. For years, the role of post-operative radiotherapy in resected pathological stage IIIA (N2) NSCLC has been controversial. However recently, two phase III RCTs have reported that adjuvant radiotherapy did not improve DFS versus placebo [6,7].

As targeted therapies, mainly tyrosine kinase inhibitors (TKIs) in NSCLC with actionable genomic alterations (AGA), and immune checkpoint blockers (ICB) in NSCLCs without AGAs, have improved the outcome in metastatic setting [8,9], these strategies have also been tested in patients with resectable early-stage NSCLC. In this review, we summarize current treatment data with these strategies and discuss challenges associated with these approaches in this setting.

## 2. Adjuvant and Perioperative Treatment in NSCLC with Actionable Oncogene Alterations

In resectable *EGFR*-mutant NSCLC, several clinical trials have reported that adjuvant treatment with first-generation EGFR TKIs improves the DFS but without meaningful benefit in OS. This could be explained by the subsequent EGFR TKI treatment post-progression in the adjuvant chemotherapy arm but also due to the lack of capacity of these agents to change the natural history of the disease, based on the limited intracranial penetration resulting in poor central nervous system (CNS) protection [10]. However, after introduction of next-generation TKIs, the role of the adjuvant-personalized approach has recently shifted. Currently, both the Food Drug Agency (FDA) and the European Medicament Agency (EMA) have approved two adjuvant-targeted TKIs, osimertinib and alectinib, for *EGFR*-mutant (osimertinib) and *ALK*-positive (alectinib) in completely resected early-stage NSCLC.

The phase III ADAURA trial evaluated in 682 patients with completely resected *EGFR*-mutant stage IB (≥3 cm)-IIIA NSCLC (7th TNM classification) the DFS benefit of osimertinib (80 mg once daily, QD) or placebo for 3 years, after optional adjuvant chemotherapy [11]. Osimertinib significantly improved the DFS in the primary endpoint population, i.e., those with a stage II-IIIA NSCLC (65.8 months vs. 21.9 months, HR: 0.23; 95% CI: 0.18–0.30) as well as in the overall population, i.e., stage IB-IIIIA (65.8 months vs. 28.1 months, HR: 0.27; 95% CI: 0.21–0.34) [12]. The magnitude of the DFS benefit with osimertinib increased with the stage, with an HR of 0.41 (0.23–0.69), HR: 0.34 (0.23–0.52), and HR: 0.20 (0.14–0.29) for stage IB, II, and IIIA, respectively [12]. Likewise, adjuvant treatment with osimertinib significantly improved the OS compared with placebo in patients with stage II–IIIA disease (HR: 0.49; 95% CI: 0.33–0.73; *p* = 0.0004 with a 5-year OS rate of 85% with osimertinib vs. 73% with placebo), as well as in the overall population (HR: 0.49; 95% CI: 0.34–0.70; *p* < 0.0001, with a 5-year OS rate of 88% with osimertinib and 78% with placebo, respectively). However, the crossover rate was low, as only 43% of patients in the control arm with progressive disease who received a subsequent treatment, received osimertinib as a subsequent treatment [13]. Of note, in the trial, the benefit in DFS and OS with osimertinib occurred regardless of the use of adjuvant chemotherapy [13]. Indeed, although the magnitude of benefit with osimertinib in terms of DFS occurred regardless of ethnicity, the OS benefit was more pronounced in non-Asian (HR: 0.33; 95% CI: 0.17–0.61) than in Asian patients (HR: 0.61; 95% CI: 0.38–0.97). Finally, adjuvant treatment with osimertinib resulted in CNS protection. In patients with stage II-IIIA disease, CNS DFS HR was 0.24 (95% CI: 0.14–0.42), with an estimated probability of observing CNS recurrence at 3 years of 2% with osimertinib versus 13% with placebo [12]. The grade ≥3 adverse events (AEs) rate was 20% with osimertinib and 13% in the placebo arm. Higher dose interruptions (24% vs. 11%), dose reductions (9% vs. 1%), and discontinuations (11% vs. 3%) occurred in the osimertinib arm than in the placebo arm [11], without a negative impact on patients’ health-related quality of life [14].

Based on the DFS data, osimertinib gained marketing authorization as an adjuvant treatment for patients with completely resected stage IB-IIIA NSCLC and a common sensitizing *EGFR* mutation (Del 19 or L858R). Ongoing studies are designed to answer additional questions for patients with resectable *EGFR*-mutant NSCLC. The ADAURA2 trial (NCT05120349) evaluates whether adjuvant TKI can benefit very early-stage NSCLC by offering osimertinib as an adjuvant treatment to resected stage IA2-IA3 NSCLC. The phase II TARGET trial (NCT05526755) addresses whether an extended duration up to 5 years of adjuvant osimertinib is beneficial in terms of DFS for common *EGFR*-mutant stage II-IIIB NSCLC. Of note, the trial also includes a cohort of patients with *EGFR*-uncommon (G719X, L861Q, and/or S768I) EGFR-TKI-sensitizing mutations to assess the benefit of adjuvant osimertinib in this setting as a secondary endpoint. The optimal treatment duration with adjuvant EGFR TKI is of relevance, as in several trials the DFS benefit with adjuvant EGFR TKI began to converge with the control arm after EGFR TKI discontinuation [13,15], suggesting that adjuvant EGFR TKI can only delay the recurrence of disease but cannot completely destroy the dormant cancer cells. Although it could be hypothesized that longer treatment duration may impact the outcome, it is not completely supported by recent clinical data. The phase III ICTAN trial randomized 251 Chinese patients with resected stage II-IIIA *EGFR*-mutant NSCLC, and after at least two cycles of adjuvant chemotherapy, were randomized to observation or to receive 12 months versus 6 months of adjuvant icotinib (a first-generation EGFR TKI). After a median follow-up of 61.4 months, 6 months of icotinib significantly improved DFS (HR: 0.41; 95% CI: 0.27–0.62; *p* = 0.000025) and OS (HR: 0.56; 95% CI: 0.32–0.98; *p* = 0.041) compared with observation. Similarly, 12 months of adjuvant icotinib compared to observation significantly improved DFS (HR: 0.40; 95% CI: 0.27–0.61; *p* = 0.000014) and OS (HR: 0.55; 95% CI: 0.32–0.96; *p* = 0.035). However, 12 months of adjuvant icotinib neither improved DFS (HR: 0.97; *p* = 0.89) nor OS (HR: 1.00; *p* = 0.99) compared with 6 months of treatment. Although the trial achieved the primary endpoint, it terminated early due to slow accrual [16].

Finally, the NeoADAURA trial (NCT04351555) compares osimertinib with or without chemotherapy versus chemotherapy alone as neoadjuvant treatment for resectable *EGFR*-mutated stage II-IIIB(N2) NSCLC, aiming to determine whether perioperative TKI can benefit patients. This is of interest as previous data with neoadjuvant osimertinib monotherapy shows very promising responses rates but limited major pathological responses (MPR, defined as ≤10% residual viable cells) or pathological completed responses (pCR) [17,18,19], both accepted as prognostic markers correlated with improved outcome [20]. Indeed, even when using neoadjuvant osimertinib resulting in high rates of response, 11% of patients did not undergo surgery after induction treatment and were converted to definitive chemoradiotherapy [17]. These data may suggest that a more intensive neoadjuvant treatment could be necessary to improve pathological endpoints that correlate with outcome.

In the phase III ALINA trial, 257 patients with completely resected, *ALK*-positive NSCLC stage IB (tumors ≥4 cm), II, or IIIA NSCLC (7th TNM classification) were randomized to alectinib (600 mg twice daily for 24 months) or to four cycles of adjuvant chemotherapy. Alectinib achieved the primary endpoint as it significantly improved the DFS compared to adjuvant chemotherapy in patients with stage II-IIIA NSCLC (HR: 0.24; 95% CI: 0.13–0.45; *p* < 0.001; with a 2-year DFS of 94% and 63%, respectively; and 94% and 64%, respectively, in the intention-to-treat population; HR: 0.24; 95% CI: 0.13–0.43; *p* < 0.001). The benefit of alectinib was generally consistent across all subgroups, and occurred regardless of disease stage, race, sex, and smoking status. The most common site of recurrence was the brain, and alectinib significantly reduced this risk, with an HR of CNS disease recurrence or death of 0.22 (95% CI: 0.08–0.58). At the data cutoff, data for OS were still immature. The majority of AEs were grade one or two, the most commonly reported AEs were increased creatine kinase levels (43.0%) and constipation (42.2%) in the alectinib group and nausea (72.5%) and decreased appetite (29.2%) in the chemotherapy group. Grade ≥3 TRAEs occurred in 18% of patients in the alectinib arm and 28% in the adjuvant chemotherapy arm, leading to dose reductions in 26% and 10% of patients, respectively, and treatment discontinuation in 6% and 13%, respectively [21]. It will be of interest to explore if the alectinib dose in the adjuvant setting should be adapted in the Japanese population as in the metastatic setting the optimal dose for alectinib was the 300 mg twice daily in this patient population [22]. This is of relevance for the lifetime chronic toxicity, as well as for the economic impact in the adjuvant setting. Altogether, these data support changing our practice in the adjuvant setting of *ALK*-positive resected NSCLC.

In the setting of *ALK*-positive NSCLC, there are ongoing clinical trials evaluating the perioperative strategy such as the NAUTIKA (NCT04302025), with multiple therapies in biomarker-selected patients with resectable stage IB-IIIA NSCLC and the ALNEO trial (NCT05015010), a phase II study assessing the perioperative strategy in patients with resectable stage III *ALK*-positive NSCLC.

## 3. Treatment Approach in Unresectable NSCLC with Actionable Oncogene Alterations

In unresectable stage III NSCLC, a post hoc analysis of the PACIFIC trial (testing durvalumab versus placebo after concurrent chemoradiotherapy) in 35 patients with *EGFR*-mutant stage III NSCLC reported no DFS benefit from durvalumab versus placebo (median DFS: 11.2 vs. 10.9 months, HR: 0.91; 95% CI: 0.39–2.13; and median OS: 46.8 vs. 43.0 months, respectively, HR: 1.02; 95% CI: 0.39–2.63), suggesting that a consolidation treatment with EGFR TKI would be more suitable [23].

Recently, the LAURA trial enrolled 216 patients with unresectable stage III NSCLC harboring a common sensitizing *EGFR* mutation initially treated with chemoradiotherapy. The trial reported significantly longer progression-free survival (PFS) with lifetime osimertinib compared to placebo as consolidation treatment after radical chemoradiotherapy (median PFS: 39.1 months versus 5.6 months, HR: 0.16; 95% CI: 0.10–0.24; *p* < 0.01) with 1-year PFS of 74% vs. 22%, respectively). The interim OS (with only 20% of maturity) did not report differences, with a 3-year OS rate of 84% with osimertinib and 74% with placebo, as 92% of patients in the control who progressed and received subsequent treatment, received osimertinib. The incidence of grade ≥3 AEs was 35% in the osimertinib group and 12% in the placebo group, and radiation pneumonitis (majority grade, one to two) occurred in 48% and 38% of patients, respectively [24]. The immature OS data with a lifetime treatment, the underperformed outcome in the control arm, and the lack of predictive biomarkers may be clinical challenges to be addressed in daily practice before worldwide acceptance of this strategy. Similarly, a small retrospective cohort of 64 patients with stage III *ALK*-positive NSCLC, consolidation ALK TKI demonstrated clinically meaningful improvement in PFS and OS over durvalumab and observation, with a better safety profile [25]. These data suggest that consolidation ALK TKI could also be appropriate in this setting. In this scenario, the phase III HORIZON-01 trial (NCT05170204) evaluates alectinib versus durvalumab as consolidation treatment after chemoradiotherapy in patients with unresectable stage III *ALK*-positive NSCLC.

## 4. Challenges in Oncogene-Driven Early-Stage NSCLC

The current challenges to be addressed with adjuvant TKI in early-stage oncogene-addicted NSCLC include the role of minimal residual disease (MRD), specifically the detection of circulating tumor DNA (ctDNA) after radical treatment, to identify patients at high risk for disease relapse that may obtain maximum benefit of the adjuvant treatments, or the optimal adjuvant treatment duration [26]. This is even more relevant in the unresectable stage III setting, as consolidation treatment with osimertinib continues until disease progression, even though an OS benefit has not yet been reported [24]. In this scenario, a prospective cohort including 278 patients with completely resected stage IA-IIIA *EGFR*-mutant NSCLC explored the longitudinal ctDNA status by digital-droplet polymerase chain reaction. Pre-surgery ctDNA was detected in 67 (24.1%) patients. Patients without ctDNA detection or those with clearance of ctDNA (76%) after surgery had significantly higher 3-year DFS rates compared to those without ctDNA clearance (3-years DFS: 83.3%, 78% and 50%, respectively, *p* = 0.02) [27]. However, in the tumor-informed MRD analysis from the ADAURA trial, performed in just one-third of the overall population, only 4% (n = 5) in the osimertinib arm and 12% (n = 13) in the placebo arm were MRD positive at baseline. Adjuvant treatment with osimertinib cleared the ctDNA in four out of five patients. During treatment, MRD detection preceded a DFS event by a median of 4.7 months across both arms. Most patients remained MRD-negative during adjuvant treatment with osimertinib, with the majority of MRD/DFS events occurring after the completion of osimertinib treatment [28]. Altogether, these data suggest that MRD positivity is a prognostic marker. If MRD becomes a standard for the daily practice, it will be relevant to define the most optimal techniques and platforms to ensure sensitive techniques to detect MRD, aiming to perform treatment decisions based on their results (intensification strategies or not). Additionally, longitudinal detection of MRD could potentially identify a subset of patients likely to benefit from extended adjuvant treatment with osimertinib.

Another challenge is to determine whether we can safely omit adjuvant chemotherapy in oncogene-driven NSCLC, considering that this treatment improves the 5-year OS in completely resected NSCLC [3]. In contrast to the ALINA trial, the NAUTIKA trial (NCT04302025) will administer adjuvant chemotherapy after induction treatment with a TKI. Furthermore, additional research is needed to investigate how co-mutations in tumor suppressor genes may impact the efficacy of adjuvant TKIs in oncogene-addicted NSCLC.

Likewise, elucidating the mechanism of acquired resistance to adjuvant TKIs, and determining the optimal treatment at progression, as well as understanding the role of TKI-rechallenge, remains unknown [10,29]. Adjuvant treatment with TKIs is being explored in tumors with other oncogene drivers such as *RET*-fusions (NCT03157128 and NCT04819100) or *MET*-deregulated NSCLCs (NCT04926831). This raises the question of whether we should conduct a separate clinical trial for each genomic alteration, an umbrella trial, or accept that the benefit of adjuvant TKIs occurs regardless of the genomic alteration subtype, as seen in metastatic disease. However, not all TKIs exhibit a strong intracranial activity, which is a significant driver of their survival benefit in the adjuvant setting, particularly in reducing the risk of brain progression.

## 5. Immune Checkpoint Blockers in the Adjuvant Setting in Early-Stage NSCLC

**Figure 1:**
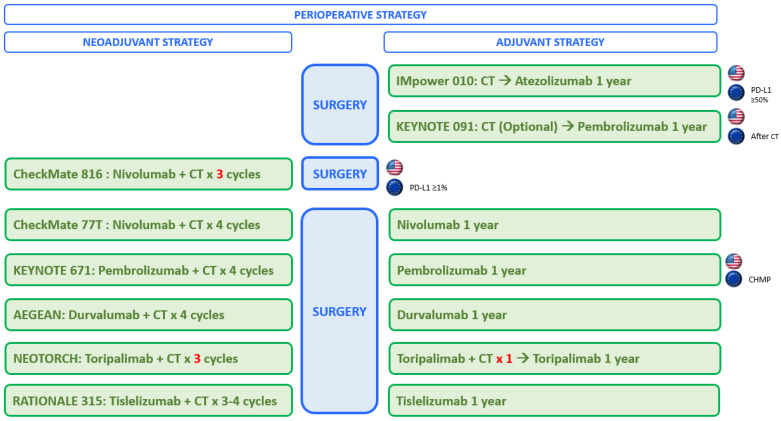
Immunotherapy strategy in patients with early-stage non-small cell lung cancer. Figure created from references [31,32,33,34,35,36,37,38].

In resectable early-stage NSCLCs, ICB have been tested and approved in the adjuvant setting (after surgery), in the neoadjuvant setting (before surgery), and in the perioperative setting (before and after the surgery, Figure 1). These new treatment strategies require important modifications to the patient treatment journey trajectory, with several clinical challenges that have to be answered in the coming years. Therefore, the International Association for the Study of Lung Cancer (IASLC) recently issued a consensus recommendation aimed at providing practical guidance for the patient’s journey, based on the latest clinical evidence [30].

Two randomized phase III clinical trials have defined the role of ICB in the adjuvant setting in completely resected stage IB (>4 cm)-IIIA NSCLC, according the 7th TNM, leading to the approval of this strategy by the health authorities [31,32]. The IMpower 010 trial randomized 1005 patients to receive adjuvant atezolizumab (1200 mg every 3 weeks) for 1 year or best supportive care after at least 1 cycle of adjuvant chemotherapy. The trial met its primary endpoint as adjuvant atezolizumab significantly improved the DFS in patients with stage II-IIIA PD-L1 ≥1% NSCLC over best supportive care (median DFS: not reached, NR vs. 35.3 months, HR: 0.66; 95% CI: 0.5–0.88; *p* = 0.004) [31]. Based on this positive primary endpoint, in 2021 the FDA approved the use of adjuvant atezolizumab in this setting. For the whole population with II-IIIA NSCLC, median DFS was 42.3 months vs. 35.3 (HR: 0.79; 95% CI: 0.64–0.96; *p* = 0.02), respectively; for the ITT it was NR vs. 37.2 months (HR: 0.81; 95% CI: 0.67–0.99; *p* = 0.04, boundary for statistical significance not crossed due to hierarchical design of the study). An exploratory analysis of patients with stage II-IIIA, according to PD-L1 expression, reported that the benefit in DFS was mainly driven by tumors with high PD-L1 expression ≥50% (HR 0.43; 95% CI: 0.23–0.68) with no DFS benefit for NSCLC with intermediate PD-L1 expression 1–49% (HR: 0.87; 95% CI: 0.60–1.26) [31]. Therefore, the EMA restricted adjuvant atezolizumab approval to tumors with high PD-L1 expression. In the first pre-specified interim analysis of OS, atezolizumab did not show survival benefit in PD-L1 ≥1% stage II-IIIA NSCLC (HR: 0.71; 95% CI: 0.49–1.03). Nevertheless, a survival benefit was shown for the subgroup of tumors with high PD-L1 expression (HR 0.42; 95% CI: 0.23–0.78) with a 5-year OS of 85% vs. 68% [39]. Whether the OS benefit with atezolizumab in the subgroup of patients whose tumors expressed PD-L1 ≥ 50% should be attributed to the impact of the very-high expression subgroup (PD-L1 ≥ 90%) or to the low percentage of treatment with ICBs at progression in the control arm, remains to be clarified.

The KEYNOTE-091/PEARLS trial randomized 1177 patients with completely resected stage IB-IIIA NSCLC treated with or without adjuvant chemotherapy to receive one year of adjuvant pembrolizumab (200 mg every 3 weeks) or placebo. Adjuvant treatment with ICB significantly improved the DFS versus placebo in the overall population (HR: 0.81; 95% CI: 0.68–0.96; with a 4-year DFS of 52% vs. 45.7%, respectively), reaching the dual primary endpoint of the study. However, in this study, adjuvant pembrolizumab did not improve the dual DFS endpoint in PD-L1 ≥50% tumors (HR: 0.83; 95% CI: 0.59–1.16; *p* = 0.13; 4-year DFS rates were 57.0% vs. 49.1%, respectively) [32,40]. To date, adjuvant pembrolizumab is approved by both the FDA and the EMA regardless of PD-L1 expression. However, EMA approval requires that patients must have undergone prior adjuvant chemotherapy, as the study’s subgroup analysis indicated that those who did not receive adjuvant chemotherapy did not benefit from adjuvant pembrolizumab [32]. To date, OS data from this study are still immature.

There are also negative results with adjuvant ICB. A recent press release reported that the phase III BR31 trial (NCT02273375) testing adjuvant durvalumab in patients with resected stage IB-IIIA NSCLC with PD-L1 expression ≥25% did not lead to statistically significant improvement in DFS compared with placebo. However, the role of adjuvant ICB is still being explored in other phase III clinical trials (ANVIL: NCT02595944, with adjuvant nivolumab, and ACCIO: NCT04267848, with adjuvant and perioperative pembrolizumab). Results of these studies may endorse the role of ICB in the adjuvant setting and define the most optimal population to receive this strategy. However, the increased acceptance of induction strategies may limit the application of solely an adjuvant approach.

## 6. Immune Checkpoint Blockers in the Perioperative Setting in Early-Stage NSCLC

As reported, systemic treatment compliance is higher when chemotherapy is given as an induction (neoadjuvant) treatment compared to adjuvant therapy [5]. Importantly, neoadjuvant systemic therapy enables for the evaluation of pathological complete response (pCR) after surgery, which is a relevant surrogate outcome for improved outcome [20].

The pivotal phase II CheckMate 159 trial showed that just two cycles of neoadjuvant nivolumab in 21 patients with resectable stage IB-IIIA NSCLC yielded a 45% MPR, including 15% of pCR [41]. With a median follow-up of 63 months, 5-year recurrence-free survival (RFS) and OS rates were 60% and 80%, respectively. At 5-year follow-up, 89% of patients with MPR were alive and disease-free [42]. Subsequently, several phase II trials have reported data with neoadjuvant ICB as monotherapy or dual ICB combinations [43,44,45]. Although all these trials reported data that compare favorably to historical trends in terms of MPR and pCR, results should be interpreted with caution due to small sample size, overall low recurrence rate, no control arm, and lack of robust predictive biomarkers.

To enhance the efficacy of neoadjuvant ICB, and based on the additive effect observed in the metastatic setting [46], neoadjuvant ICB plus chemotherapy and perioperative ICB strategies have also been explored in resectable early-stage EGFR/ALK-wildtype NSCLC. Table 1 and Figure 1 summarize the most relevant randomized phase III clinical trials testing neoadjuvant chemotherapy plus ICB versus chemotherapy alone [33], and clinical trials assessing the perioperative strategy (ICB before and after the surgery) versus chemotherapy alone [33,34,35,36,37,38]. Briefly, all of these trials have reported the ICB arm achieving significantly higher pCR compared with chemotherapy alone (~20% vs. ~5%, respectively), and longer event-free survival (EFS), reducing the risk of recurrence by approximately 40%. One trial has also reported significant improvement in OS (HR: 0.72; 95% CI: 0.56–0.93; *p* = 0.00517) [35,47]. For other phase III RCTs testing perioperative ICB, OS data are not yet mature. However, up to 20% of patients treated with an induction treatment of chemotherapy with or without ICB do not undergo surgery due to several reasons [43].

Currently, the FDA approved the neoadjuvant approach with nivolumab, whereas the EMA restricted the approval of neoadjuvant nivolumab to those tumors with PD-L1 ≥1% NSCLC. However, the FDA and the EMA approved the perioperative strategy with pembrolizumab. Despite prescribing limitations, a recent meta-analysis reported that a benefit of chemoimmunotherapy in the early-stage NSCLC occurred regardless of the PD-L1 expression, with benefit in EFS even in PD-L1 < 1% tumors (HR: 0.74; 95% CI: 0.62–0.89), and regardless of stage of the disease (stage II, HR: 0.71; 95% CI: 0.55–0.92; and stage III, HR: 0.54; 95% CI: 0.48–0.62), and regardless the type of platinum chemotherapy [48].

**Table 1:** Summary of phase III clinical trials testing neoadjuvant and perioperative strategy in resectable early-stage non-small cell lung cancer.

Study	Perioperative /Neoadjuvant	N	Percentage of PD-L1 > 1%	Percentage of Stage III %	Percentage of Surgery	pCR CT-ICB vs. CT	EFS HR [95% CI, *p*]	OS HR [95% CI, *p*]
**CheckMate 816** [33,49]	Neoadjuvant	358	50%	63%	83%	24% vs. 2.2%	0.66 [0.49–0.9]	0.71 [0.47–1.07, *p* = 0.045] *
**CheckMate 77T** [34]	Perioperative	461	56%	64%	77%	25% vs. 5%	0.58 [0.42–0.81, *p* < 0.00025)	NR
**KEYNOTE671** [35,47]	Perioperative	797	65%	70%	82%	18% vs. 4%	0.59 [0.48–0.72]	0.72 [0.56–0.93, *p* < 0.01)
**AEGEAN** [36]	Perioperative	802	67%	71%	81%	17% vs. 4%	0.68 [0.53–0.88, *p* < 0.01]	NR
**NEOTORCH** [37]	Perioperative	501	66%	100%	82%	25% vs. 1.0%	0.40 [0.28–0.57, *p* < 0.01]	0.62 [0.38–1.0, *p* = 0.05]
**RATIONALE 315** [38]	Perioperative	453	58%	58%	84%	41% vs. 5.7%	0.56 [0.40–0.79, *p* <0.01]	0.62 [0.39–0.98, *p* = 0.02]

pCR: pathological complete response; EFS: event free survival; OS: overall survival; HR: hazard ratio; 95% CI: 95% confidence interval; NR: not reported; CT chemotherapy; ICB: immune checkpoint blockers (CheckMate 816 and CheckMate 77T: nivolumab; KEYNOTE671: pembrolizumab; AEGEAN: durvalumab; NEOTORCH: toripalimab; RATIONALE 315: tislelizuamb); * significant boundary for OS was not met (*p* = 0.0164) at this interim analysis.

Of note, long-term data with the induction nivolumab plus chemotherapy are already available. In the CheckMate 816 trial, the 4-year EFS improved 11% in the nivolumab arm compared to chemotherapy alone (4-years EFS: 49% vs. 38%; HR: 0.66; 95% CI: 0.49–0.90), with a median EFS of 44 months vs. 18.4 months, respectively), with a non-significant benefit according to boundary criteria for OS (4-year OS: 71% vs. 58%; HR: 0.71; 95% CI: 0.47–1.07; *p* = 0.045) [49].

The benefit of the induction or perioperative strategy in early-stage NSCLC occurs regardless of the lymph node N2 status. An exploratory analysis from the CheckMate 77T trial evaluating perioperative nivolumab showed that over half of the patients with stage III disease had nodal downstaging with nivolumab, and the majority downstaged to ypN0. Indeed, in the trial, multi-station N2 versus single-station N2 tumors achieved better efficacy with the chemoimmunotherapy combination approach in terms of pCR (29% vs. 19%) and EFS from definitive surgery (HR: 0.23 [0.09–0.58] for multi-station N2 and HR: 0.40 [0.20–0.78]) [50], suggesting that multi-station N2 is not a limitation for induction treatment. Similarly, in the AEGEAN trial evaluating perioperative strategy with durvalumab, 49.5% of patients had baseline positive N2 nodal status. Perioperative durvalumab plus neoadjuvant chemotherapy compared to neoadjuvant chemotherapy alone prolonged the EFS (HR: 0.63; 95% CI: 0.43–0.90) and increased the rates of pCR (16.6% vs. 4.9%; difference, 11.7% (95% CI: 5.6–18.4)), with a benefit similar to that observed in the intention to treat population, with no adverse impacts in surgical outcomes [51]. Altogether, these data support the induction or perioperative strategy in resectable early-stage NSCLC as a standard treatment approach in daily practice.

## 7. Challenges with Adjuvant and Perioperative ICB Strategy

**Figure 2:**
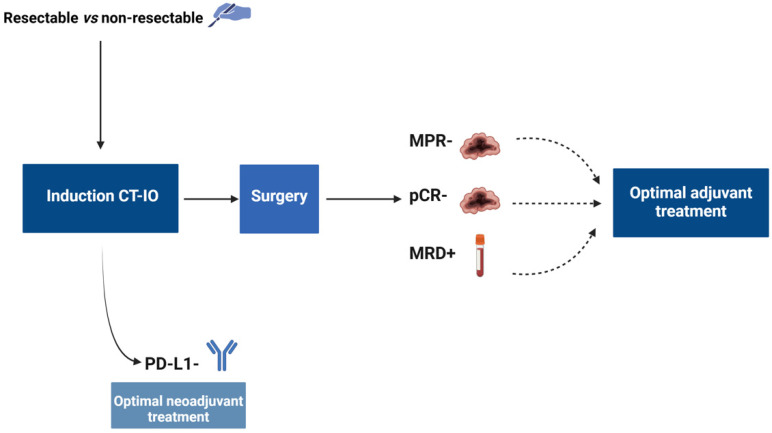
Challenges with immune checkpoint blockers and targeted therapies in early-stage non-small cell lung cancer (Figure created by Biorender.com).

Despite the therapeutic shift with ICB in early-stage NSCLC, several clinical challenges remain open (Figure 2). Similar to oncogenic addicted tumors, the role of MRD in deciding to prescribe adjuvant ICB is a relevant clinical challenge, particularly for patients initially treated with induction chemoimmunotherapy. An exploratory analysis of the IMpower 010 trial [52] showed that post-resection ctDNA positivity was strongly prognostic and correlated with disease stage, nodal status, and *EGFR* status. Although the absolute DFS benefit with adjuvant atezolizumab increased in MRD-positive patients, benefit was seen regardless of post-resection MRD status (HR for DFS 0.61 and 0.72 for MRD-positive and MRD-negative patients, respectively), thus revealing the absence of a predictive impact of MRD in this setting. As MRD positive is a strong negative prognostic marker, and correlates with shorter DFS and OS [53], an additional question is whether treating patients at the time of molecular progression (MRD positive) improves survival compared to treating patients with molecular disease once radiological progression is observed.

Data from trials evaluating the perioperative strategy suggest that although the benefit of adjuvant ICB occurred regardless of the pCR status, this benefit is more pronounced in tumors without pCR [34,35,37]. In the future, composite endpoints such as MRD and pCR status may construct a risk stratification model and identify the population who could avoid adjuvant treatment after induction therapy, and the population who would benefit the most from adjuvant treatment. Likewise, it will be relevant to determine the best approach for patients who are MRD-positive and do not achieve pCR: continuing with the same adjuvant ICB used in the neoadjuvant treatment, intensifying adjuvant treatment with dual ICBs, or exploring other strategies such as adjuvant vaccines.

Moreover, clinical trials with induction immunotherapy have excluded tumors with *EGFR/ALK* alterations; however, it is unknown if selected oncogene drivers, such as *KRAS* or *BRAF* mutant tumors, should also be excluded from induction treatment with ICB in the coming future because *KRAS* mutated tumors, especially, can respond to ICB as reported in the metastatic setting.

With the current induction ICB strategy, only 20% of tumors achieve a pCR. Therefore, another challenge is the role of intensifying the entire perioperative strategy to improve the outcomes, as being explored in the ongoing NEOCOAST2 trial (NCT05061550). Similarly, the best treatment approach for patients who do not proceed to surgery after induction chemoimmunotherapy remains unknown. An important additional challenge is the potential predictive biomarkers for efficacy. The benefit of induction treatment increases with a higher PD-L1 expression [48], but the optimal approach to boost activity in PD-L1-negative tumors remains unknown. Although some studies report better pathological outcomes with dual ICB in PD-L1-negative tumors [44], and there are data supporting that neoadjuvant nivolumab plus ipilimumab is feasible [54], the prospective evaluation of this approach in PD-L1-negative tumors has not been explored. Similarly, the question regarding the optimal duration of adjuvant ICB therapy to balance efficacy and long-term toxicity remains unanswered, particularly in patients who have received neoadjuvant ICBs plus chemotherapy.

Finally, to reduce both patients’ toxicity and financial toxicity, it is essential to assess the optimal number of cycles of induction chemoimmunotherapy treatment. An exploratory analysis from the CheckMate 77T trial showed that patients experienced improved efficacy with perioperative nivolumab versus placebo regardless of whether they completed four or fewer cycles. The EFS rate and proportion of pCR and MPR were similar regardless four cycles or less than four cycles of induction chemoimmunotherapy [55].

All cases of early-stage NSCLC must be discussed in a multidisciplinary tumor board to define the best treatment approach. Although phase III clinical trials testing perioperative or neoadjuvant chemoimmunotherapy include only resectable early-stage NSCLC, the definition of resectable disease is not homogeneous, especially for stage III NSCLC. Indeed, any of the prospective trials dealing with perioperative management described the surgical procedure and all these trials inappropriately evaluated the quality of the surgery, and have not applied the surgical quality control based on new criteria [56]. Surgery should follow the deep advancement of perioperative therapies. To partially overcome this scenario, a recent multisociety EORTC-LCG consensus has proposed a definition of resectable stage III NSCLC to be applied in future clinical trials [57].

Based on the clinical meaningful benefit reported with the induction chemoimmunotherapy strategy in resectable early-stage NSCLC, several clinical trials have been launched to assess the feasibility and potential benefits of surgical conversion with neoadjuvant immune-based therapy in borderline resectable early-stage or unresectable stage III NSCLC. The phase II ongoing MDT-BRIDGE trial (NCT05925530) assesses in resectable or borderline resectable stage II-IIIB NSCLC two cycles of neoadjuvant durvalumab and chemotherapy followed by either surgery and adjuvant durvalumab or chemoradiotherapy and consolidation durvalumab according to the resectability criteria after induction assessed by a multidisciplinary team. Similarly, in unresectable stage III NSCLC, a phase II clinical trial has evaluated the induction treatment with SHR-1701 (a bifunctional agent composed of an IgG4 monoclonal antibody targeting PD-L1 fused with extracellular domain of the TGF-beta II receptor) with or without chemotherapy. After induction treatment, patients were assessed in a multidisciplinary tumor board to evaluate surgery or definitive chemoradiotherapy. Surgical conversion occurred in 25% of patients (27/107) initially enrolled, reporting a pCR of 26%. The median EFS for patients receiving surgery was NR and it was 24.2 months for those treated with definitive radiotherapy, and the 18-month EFS rate was 74.1% in resected patients versus 57.3% in patients receiving radiotherapy, respectively [58]. These data appear more promising than data reported in the PACIFIC trial [59], and suggest that surgical conversion is feasible and associated with better outcomes. Larger prospective clinical trials are needed to confirm this approach as practical strategy in the daily clinic. However, results of the trial testing SHR-1701 have established a treatment paradigm involving surgery in the management of unresectable stage III NSCLC.

## 8. Conclusions

New treatment strategies have shifted the treatment landscape of early-stage NSCLC. However, it is important determine an accurate stage of the disease (including PET-CT, brain MRI, or brain CT-scan) and perform a molecular diagnosis before making any treatment decision to ensure a most effective treatment in early-stage.

Although perioperative immunotherapy in early-stage NSCLC has changed the standard of care for patients with resectable NSCLC, several relevant clinical questions remain unresolved, such as the role of adjuvant ICB after an induction approach and the optimal duration of the post-operative treatment. The use of adjuvant-targeted TKIs for actionable oncogene NSCLC raise the importance of molecular testing in an early-stage setting, aiming to define the optimal therapeutic approach for each patient. All of these new treatment strategies, along with other health measures such as increased screening programs, selection of predictive markers, and residual disease detection assays, hold great potential to continue to improve outcome in this setting. However, the next challenges will be to avoid excessive treatment and exposure to unnecessary toxicity, and to try to minimize healthcare costs to ensure that all patients can access effective therapies in the perioperative setting.
